# Clinical Impact of Colonization with Carbapenem-Resistant Gram-Negative Bacteria in Critically Ill Patients Admitted for Severe Trauma

**DOI:** 10.3390/pathogens11111295

**Published:** 2022-11-04

**Authors:** Giancarlo Ceccarelli, Francesco Alessandri, Sonia Moretti, Alessandra Borsetti, Maria Teresa Maggiorella, Silvia Fabris, Alessandro Russo, Franco Ruberto, Daniele De Meo, Massimo Ciccozzi, Claudio M. Mastroianni, Mario Venditti, Francesco Pugliese, Gabriella d’Ettorre

**Affiliations:** 1 Department of Public Health and Infectious Diseases, Sapienza University of Rome, 00185 Roma, Italy; 2 Azienda Ospedaliero-Universitaria Policlinico Umberto I, 00161 Rome, Italy; 3 M.I.T.O. Group (Infectious Diseases in Traumatology and Orthopedics Surgery), Policlinico Umberto I, University Hospital, 00161 Rome, Italy; 4 Intensive Care Unit, Department of General Surgery Surgical Specialties and Organ Transplantation “Paride Stefanini”, Sapienza University of Rome, 00185 Rome, Italy; 5 National HIV/AIDS Research Center, Istituto Superiore di Sanità, 00161 Rome, Italy; 6 Medical Statistics and Epidemiology Unit, Campus Bio-Medico University of Rome, 00128 Roma, Italy; 7 National Center for Control and Emergency Against Animal Diseases and Central Crisis Unit—Office III, Directorate General for Animal Health and Veterinary Drugs, Italian Ministry of Health, 00153 Rome, Italy; 8 Unit of Infectious and Tropical Diseases, Department of Medical and Surgical Sciences, “Magna Graecia” University, 88100 Catanzaro, Italy; 9 Department of Anatomical Histological Forensic Medicine and Orthopedic Science University of Rome, 00161 Rome, Italy

**Keywords:** colonization, infection, rectal swab, Acinetobacter, Klebsiella, Enterobacteriaceae, carbapenem, carbapenem-resistant Gram-negative bacteria, polytrauma, trauma, ICU

## Abstract

Multidrug-resistant (MDR) Gram-negative bacteria (GNB) have raised concerns as common, frequent etiologic agents of nosocomial infections, and patients admitted to intensive care units (ICUs) present the highest risk for colonization and infection. The incidence of colonization and infection in trauma patients remains poorly investigated. The aim of this study was to assess the risk factors for Carbapenem-resistant (CR)-GNB colonization and the clinical impact of colonization acquisition in patients with severe trauma admitted to the ICU in a CR-GNB hyperendemic country. This is a retrospective observational study; clinical and laboratory data were extracted from the nosocomial infection surveillance system database. Among 54 severe trauma patients enrolled in the study, 28 patients were colonized by CR-GNB; 7 (12.96%) patients were already colonized at ICU admission; and 21 (38.89%) patients developed a new colonization during their ICU stay. Risk factors for colonization were the length of stay in the ICU (not colonized, 14.81 days ± 9.1 vs. colonized, 38.19 days ± 27.9; *p*-value = 0.001) and days of mechanical ventilation (not colonized, 8.46 days ± 7.67 vs. colonized, 22.19 days ± 15.09; *p*-value < 0.001). There was a strong statistical association between previous colonization and subsequent development of infection (OR = 80.6, 95% CI 4.5–1458.6, *p*-value < 0.001). Factors associated with the risk of infection in colonized patients also included a higher Charlson comorbidity index, a longer length of stay in the ICU, a longer duration of mechanical ventilation, and a longer duration of treatment with carbapenem and vasopressors (not infected vs. infected: 0(0–4) vs. 1(0–3), *p* = 0.012; 24.82 ± 16.77 vs. 47 ± 28.51, *p* = 0.016; 13.54 ± 15.84 vs. 31.7 ± 16.22, *p* = 0.008; 1.09 ± 1.14 vs. 7.82 ± 9.15, *p* = 0.008). The adoption of MDR-GNB colonization prevention strategies in critically ill patients with severe trauma is required to improve the quality of care and reduce nosocomial infections, length of hospital stay and mortality.

## 1. Introduction

Healthcare-associated infections (HCAIs) have a higher incidence in trauma centers worldwide than in general hospitals [1]. The type and severity of trauma could influence the risk of infection, but trauma per se is not an independent risk factor for infection [2,3]. In trauma patients, the high need for diagnostic and therapeutic procedures, such as mechanical ventilation, surgery, and renal replacement therapy, increase the risk of HCAIs [4]. Recent studies of traumatized military and civilian war victims transferred to European countries for treatment have reported a high incidence of multidrug-resistant (MDR) infections. Predominant colonization by extended-spectrum beta-lactamases (ESBLs), carbapenem-resistant enterobacteria, non-fermentative Gram-negative bacteria (GNB), and methicillin-resistant *Staphylococcus aureus* (MRSA) has been reported [5,6]. Infectious complications are still well-recognized determinants of late mortality after severe traumatic injuries [7]. Several studies have been carried out to investigate the epidemiology of nosocomial infections among trauma patients, identifying specific risk factors for sepsis [8,9,10,11]. Furthermore, sepsis occurring in trauma patients has been shown to be an independent risk factor for a poor outcome [2,9]. Over the last decades, MDR-GNB have raised concerns as frequent etiologic agents of nosocomial infections, and patients admitted to intensive care units (ICUs) present the highest risk for colonization and infection [12]. From a public health point of view, infections sustained by carbapenem-resistant (CR)-GNB are considered particularly threatening because of their frequent involvement in nosocomial outbreaks and the risk of global spread. In particular, the levels of CR-*Enterobacterales* and CR-*Acinetobacter baumannii* (*Ab*) have reached hyperendemic levels in Italian hospitals, posing a major public health threat to the country [11]. Italy is one of the countries with the highest level of antibiotic resistance in Europe. If the current trends of CR in GNB are not reversed, key medical interventions will be compromised in the near future [13,14]. 

Currently, the risk factors contributing to the emergence of MDR-GNB colonization and the clinical outcomes related to colonization remain unclear; thus, the aim of this study was to evaluate risk factors for CR-GNB colonization and the clinical impact of colonization acquisition in patients with severe trauma admitted to the ICU in a CR-GNB hyperendemic country. This topic, in fact, remains poorly investigated at the moment to the best of our knowledge.

## 2. Materials and Methods

### 2.1. Study Design and Endpoints 

This was a retrospective, observational study conducted on data from the healthcare-associated infection (HAI) surveillance system active in the ICU of the Umberto I University Hospital of Rome (Italy). 

The primary endpoint was to determine the CR-GNB colonization rate in critically ill patients admitted to the ICU for severe trauma. Secondary endpoints were: (1) the rate of infections related to colonization, and (2) the death rate among those colonized in the ICU and those never colonized. 

The objective of our analysis was also to evaluate: (1) the mean time spent in the ICU among patients who acquired CR-GNB colonization in the ICU and those who did not; (2) the probability of being free from colonization after 1, 5, 10, 15, and 20 days; (3) the risk of CR-GNB colonization associated with ICU care procedures; (4) the rate and mean time spent on antibiotic treatment among those colonized and those not colonized in the ICU; (5) the rate and mean time spent on procedures related to the ICU among colonized patients who were or were not infected; and (6) the rate and mean time spent on antibiotic treatment among colonized patients who were or were not infected.

### 2.2. Recruitment of Patients, Population, and Setting

The study cohort included patients admitted to the ICU with a severe trauma between 1 January 2019 and 31 December. Inclusion criteria were an age ≥ 16 years, length of stay (LOS) in the ICU longer than 48 h, an Injury Severity Score (ISS) >15, and infection due to CR-GNB present at admission. Exclusion criteria were burn injuries, pregnancy, and multiple CR-GNB colonizations at admission. Patients colonized at admission with one CR-GNB were included in the study due the possibility of developing a new colonization sustained by a different CR-GNB. 

The Umberto I University Hospital of Rome is a highly specialized trauma center (HSTC) located in the central area of Lazio (Italy) and equipped with a devoted ICU. Umberto I University Hospital is part of the Lazio region’s Major Trauma Network [10].

### 2.3. Clinical and Microbiological Data Collection

Anamnestic and clinical data were obtained from the electronic files of patients and the nosocomial infection surveillance system database. Age; gender; comorbidities; the Charlson comorbidity index (CCI); mechanism of traumatic injury; the Acute Injury Scales (AISs) and ISS; date of admission; days since trauma date to ICU admission; the Simplified Acute Physiology Score II (SAPS II) at ICU admission; the need for mechanical ventilation, tracheostomy, chest drain, or surgery performed before or during ICU stay; the use of systemic antimicrobial agents; ICU length of stay (LOS); and mortality data were recovered. 

Microbiological data were obtained from the electronic archive of the hospital microbiology laboratory. 

Two outcome assessors (A.R. and G.G.) were independently involved in retrieving data from the online systems. In the case of nonconcordance of the data collected, a third person (G.C.) was delegated to check and judge the discrepancy.

### 2.4. Definitions 

The International Classification of Diseases (ICD-9) was used to classify major trauma events: only patients with a diagnostic code ICD-9-CM 800–959.9 were enrolled. Subjects with a diagnostic code from 940 to 949 were excluded as their trauma was related to burn, and therefore, not suitable for analysis [15]. A major trauma (or polytrauma) was defined as the Injury Severity Score being greater than 15. 

Carbapenem resistance, being intrinsic or acquired, was defined according to the 2015 CDC definitions for CR-*Enterobacterales*; CR-*Ab* and CR-*Pseudomonas aeruginosa *(*Pa*) were defined as isolates resistant to imipenem or meropenem as per the Clinical and Laboratory Standards Institute (CLSI) protocols [16,17].

CR-GNB colonization was defined as the presence of these microorganisms cultured from microbiology specimens without evidence of tissue invasion or inflammation at that body site. CR-GNB infection was defined as the invasion of the body tissues by these pathogens, resulting in disease [18]. 

The National Healthcare Safety Network protocol of the Centers for Disease Control and Prevention was adopted for the diagnostic criteria for the identification of HCAIs [19]. 

Microbiological screening was performed in all patients at ICU admission and then weekly during the whole ICU stay to detect colonization with CR-GNB: a culture of rectal swab specimens was used as the surveillance technique to detect colonization with multidrug-resistant bacteria (CR-*Enterobacterales*; CR-*Ab* and CR-*Pa*). Moreover, it was also performed in the presence of clinical events suggestive of the onset of a new infection. All isolates were evaluated for susceptibility to carbapenems by the disk diffusion method according to CLSI protocols. Isolates that showed resistance to either imipenem, meropenem, or ertapenem were considered CR-GNB. All CR-GNB isolates were screened for the presence of carbapenemase genes via a polymerase chain reaction (PCR) using specific primers. The detailed methodology of the surveillance system adopted in the ICU was described elsewhere [20]. 

### 2.5. Ethics 

The Institutional Ethics Board of the Umberto I University Hospital of Rome approved the study protocol (reference number: 6036/2020, 10.28.2020). All the patients included in the research or their caregivers signed an informed consent for the participation in the observational studies. The Strengthening the Reporting of Observational Studies in Epidemiology (STROBE) guidelines were followed to create the study.

### 2.6. Statistical Analysis

A descriptive analysis was performed to summarize the characteristics of the study population. Factors potentially associated with the occurrence of colonization were studied, excluding patients who obtained a positive rectal swab and were colonized at admission. Moreover, among all the colonized patients we explored the possible factor associated with CR-GNB infection. In case of missing data, patients were excluded from the statistical analysis. Continuous variables were compared with Student’s t-test where normally distributed; the Injury Severity Score and not normally distributed variables were compared by the Mood median test, whereas difference in proportions were studied with the Z-test and chi-square test. The normality distribution was assessed by using the Shapiro–Wilk test. We studied risks via the odds ratio (OR), focusing on the risk of colonization and risk of infection, and where necessary, univariate logistic regression models were built to determine how factors were associated with each specific outcome; to understand if demographic and clinical characteristics could affect proven risk factors, we computed multiple logistic regressions. Where appropriate, we used the Haldane–Anscombe correction. A survival analysis was performed to study the time free from CR-GNB colonization. We considered *p*-values < 0.05 statistically significant.

## 3. Results

### 3.1. Characteristics of the Cohort 

Fifty-four patients fulfilled the criteria to be included in the study. Table 1 summarizes the characteristics of the overall enrolled patients. 

### 3.2. CR-GNB Colonizations

Overall, 28 patients out of the 54 enrolled were colonized by CR-GNB. Assessing the primary endpoint, 7 patients were already colonized at ICU admission (12.96%) and 21 patients developed a new colonization during the ICU stay (38.89%). Characteristics of colonized and not-colonized patients are showed in Table 1. All CR-GNB colonization consisted of CR-*Ab* and/or CR-*Klebsiella pneumoniae *(*Kp*). 

**Figure 1:**
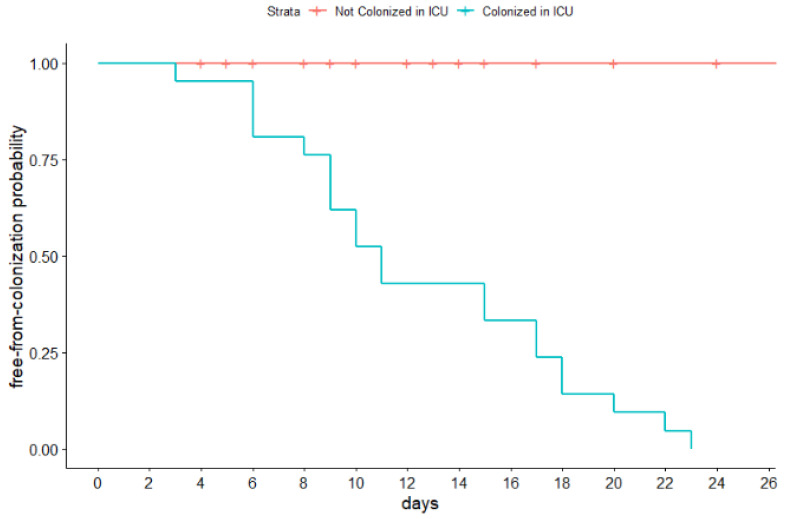
Kaplan–Meier free-from-colonization curves.

We found a statistically significant difference between patients colonized in the ICU and those not colonized in terms of ICU length of stay (not colonized, 14.81 ± 9.1 days vs. colonized, 38.19 ± 27.9 days; *p*-value = 0.001; power = 0.98): each day spent in the ICU increased the risk by 1.09 (95% CI 1.03–1.16, *p*-value = 0.003, pseudoR^2^ = 0.26). The mean time of colonization from admission to the ICU was 12.52 (5.75) days. We evaluated the time free from colonization, excluding patients colonized at admission. The results are summarized in Figure 1 and Table S1.

Moreover, when we evaluated the risk of CR-GNB colonization associated with ICU care procedures, we found a statistically significant difference in the days on mechanical ventilation (not colonized, 8.46 ± 7.67 days vs. colonized, 22.19 ± 15.09 days; *p*-value < 0.001): each day spent on mechanical ventilation increased the risk by 1.11 (95% CI 1.03–1.19, *p*-value = 0.002, pseudoR^2^ = 0.22).

Finally, we found a statistical difference between patients colonized in the ICU and not colonized in the ICU in terms of antibiotics use. In particular, the duration of antibiotic administration (not colonized, 6.65 ± 3.3 days vs. colonized, 9.76 ± 4.54 days; *p*-value = 0.013) and, specifically, the previous treatment with piperacillin/tazobactam (not colonized, 4 (15.38%) patients vs. colonized, 12 (57.14%) patients; *p*-value = 0.007) and carbapenem (not colonized, 0 vs. colonized, 6 (28.57%) patients; *p*-value = 0.013) was significantly associated with an increased risk of subsequent CR-GNB colonization. Moreover, the duration of carbapenem therapy was also associated with the colonization risk (not colonized, 0 ± 0 vs. colonized, 1.05 ± 1.94 days; *p*-value = 0.022). 

For each day of antibiotic use, the risk increased by 1.23 (95% CI 1.03–1.47, *p*-value = 0.018, pseudoR^2^ = 0.11); patients treated with piperacillin/tazobactam had an OR = 7 (95% CI 1.77–27.78, *p*-value = 0.004) and carbapenem-treated patients had an OR = 22.22 (95% CI 1.17–422.07, *p*-value = 0.006) compared to patients who did not take the specific antibiotic under evaluation.

### 3.3. Infections Due to CR-GNB

Investigating the secondary endpoints, we also evaluated factors related to the emergence of infections in colonized patients. We found a strong statistical association between previous colonization and the subsequent development of infection (chi-square *p*-value < 0.001; OR = 80.6 (95% CI 4.5–1458.6, *p*-value < 0.001)). 

In particular, both the colonization observed on admission and the colonization acquired during ICU hospitalization were significantly associated with the subsequent detection of a related infection (both *p*-values < 0.001; OR = 116.6 (95% CI 4.9–2782.1, *p*-value < 0.001) and OR = 69.7 (95% CI 3.7–1295.9, *p*-value < 0.001), respectively). 

Considering all colonized patients, we compared who developed infections and who did not: we found significant differences in terms of the Charlson comorbidity index (*p*-value = 0.012), time spent in the ICU (*p*-value = 0.016), time spent on mechanical ventilation (*p*-value = 0.008), and the number of days with the support of vasopressors (*p*-value = 0.008); results are summarized in Table 2. 

For each day in the ICU, the risk of being infected increased by 1.05 (95% CI 1.001–1.1, *p*-value = 0.046, pseudoR2 = 0.15), and for each day spent on mechanical ventilation, by 1.08 (95% CI 1.01–1.15, *p*-value = 0.019, pseudoR2 = 0.21). Moreover, we found a significant difference in terms of days of carbapenem use (*p*-value = 0.042); details are reported in Table 2. 

### 3.4. In-ICU Related Mortality

Finally, as a further secondary endpoint, we investigated the impact of colonization on in-ICU related mortality. Overall, sixteen patients died in the ICU (29.63%), with no differences between colonized (32.14%) and not-colonized (26.92%) patients (*p* value = 0.903).

## 4. Discussion

Several studies have been performed to assess the incidence and risk factors for MDR-GNB colonization in ICUs and to determine the clinical impact [21,22]. However, to the best of our knowledge, few works have focused on MDR-GNB acquisition in trauma patients, and evidence on the epidemiology and the clinical impact of CR-GNB colonization in severely traumatized patients admitted to ICUs is scant [2,23].

To correctly understand our epidemiological results, it is crucial to underline that the study setting was in Italy, a CR-GNB hyperendemic European region, in the pre-COVID-19 era [24]. The high incidence of CR-GNB colonization reported in ICU patients (overall 51.9%) appears to be particularly alarming if we observe that 12.9% out of the 54 patients enrolled were already colonized at the time of ICU admission. About this latest, impressive epidemiological data, it should be noted that patients enrolled were quickly admitted to the ICU after their first surgical and resuscitation support in the emergency room. We are not able to report the exact moment of colonization, whether it took place in the pre-trauma phase or in the emergency room (ER); however, it should be emphasized that first-aid procedures in the ER have the same infectious risk as those in the ICU and require the same attention to avoid possible MDR colonization. 

Regarding the population enrolled, it is important to note that it was, on average, young and with a low level of comorbidity, as evidenced by the CCI (Table 1): this fact should be taken into consideration, especially in the outcome assessment. Otherwise, all patients were clinically in serious condition following severe polytrauma with an ISS > 15 and required resuscitation support with mechanical ventilation. 

In our setting, the CR-GNB colonization rate was not related to the severity of illness expressed by the SAPS-II, a greater severity of the trauma evaluated in the ISS, and the number of comorbidities (CCI). It was associated with days of mechanical ventilation and, consequently, with the length of stay in the ICU. Moreover, the time of exposition to intensive respiratory care was found to be an independent risk factor for CR-*Ab* and -*Kp* colonization, with an increase in the colonization risk of 1.1 times per day. In the same way, for each day of ICU hospitalization, the risk of CR-GNB colonization was increased 1.09 times. Interestingly, after 10 days of an in-ICU stay, about 50% of the total colonizing patients had already acquired at least one CR-GNB. The higher probability of acquiring CR-GNB during a longer ICU stay could be due to prolonged exposure to the risk of transmission of CR-GNB between patients via healthcare workers, medical devices, or ICU environments [25]. This hypothesis was supported by several studies which showed that CR-GNB isolates from colonized patients were clonally related to those present on other patients, medical devices, or in hospital environments [26].

Antibiotic use was also a risk factor for CR-GNB colonization in our cohort of polytrauma patients: in particular, the overall days of antibiotic administration and, specifically, the exposure to piperacillin/tazobactam and especially to carbapenem was significantly associated with an increased risk of subsequent colonization. 

Previous studies assessing the epidemiology of CR-GNB separately analyzed the risk factors for CR-*Ab* and -*Kp* colonization in in-hospital patients [21,22]. A recent meta-analysis of CR-Kp epidemiology identified the use of carbapenems as the second most important risk factor for colonization, after the use of medical devices, with an OR of 4.01 [27]. This latter variable was not analyzed in our study because, due to the setting being an ICU, all the patients had been frequently submitted to arterial and central venous catheterization, urinary catheterization, and invasive airway management. Different studies analyzed MDR Ab colonization risk factors, identifying age, comorbidities, previous antibiotic use (including carbapenems), medical devices, previous healthcare exposure, admission with trauma, blood transfusion, low socioeconomic status, and others as variables independently associated with the outcome [28,29].

The clinical impact of colonization might be overly high, as suggested by the fact that 60.7% of CR-GNB-colonized subjects developed at least one ICU-acquired infection due to the same etiologic agents. When we analyzed the risk of infection in our cohort, we observed that a previous colonization was a strong predictor, particularly in patients with colonization at ICU admission. This significant risk could be explained by a greater likelihood of microbial translocation related to the trauma: in fact, recent studies indicate that the gut microbiome is altered early following traumatic injury, compromising the intestinal epithelial barrier and decreasing gut perfusion and inflammation [30].

Moreover, in colonized patients we observed that this risk was related with a higher CCI, time spent in ICU, the support of mechanical ventilation, days of treatment with carbapenem, and vasopressors. Interestingly, we did not observe a significant impact of CR-GNB colonization on in-ICU related mortality. We were inclined to justify the data on mortality based on the characteristics of the population enrolled: low levels of comorbidity and a young age can be considered overall protective factors in relation to the risk of death, identifying substantially “healthy patients” if trauma is excluded. 

This study has a number of limitations. Firstly, the small sample size does not allow for the construction of a predictive model, and despite the statistical evidence, the 95% CI is too wide to be clinically interpreted. The retrospective nature of the enrolment, conducted in a single center (even if the hospital is a large hub specialized in polytrauma management), may have influenced the characteristics of the polytrauma population enrolled: in particular, the young mean age of the cohort, characterized by few comorbidities, may have positively influenced some outcomes such as mortality. Finally, the study was conducted in a CR-GNB hyperendemic country: these factors may have represented biases for the generalization of the results. Therefore, it is desirable to carry out large studies that can provide external validation for the results presented here. Despite the aforementioned biases, the findings of this study have important public health implications for policy makers. In particular, we observed that the risk of colonization was related to the length of stay in the ICU: this factor should lead to strengthening the trauma networks by creating dedicated paths in order to optimize and reduce hospitalization times to a minimum.

## 5. Conclusions

Considering the results of this study and the evidence coming from the previous literature data, strategies directed toward MDR-GNB colonization prevention in patients admitted with severe trauma to the general ICU might be effective at improving the quality of care and reducing hospital-acquired complications, hospital length of stay, and mortality. 

On the other hand, it would be very interesting to confirm our results with prospective multicentric studies and to further assess independent risk factors for the development of CR-GNB-sustained hospital-acquired infections in trauma patients colonized with these agents, an issue not specifically evaluated in our work.

## Figures and Tables

**Table 1:** Characteristics of the overall enrolled patients, including the subjects not colonized and colonized.

	Total n = 54 (100%)	Not Colonized n = 26 (48.1%)	Colonized n = 28 (51.9%)	*p*-Value
Sex M—n (%)	44 (81.49)	21 (80.77)	23 (82.14)	1
Age—mean (sd)	47.72 (20.25)	43.38 (19.69)	51.75 (20.27)	0.13
Charlson comorbidity index— median (min–max)	1 (0—5)	0 (0–5)	1 (0–4)	0.634
ISS—mean (sd)	31.42 (10.87)	32 (12.4)	30.9 (9.42)	0.697
Head and neck AIS— median (min–max)	3 (0–5)	3 (0–5)	3 (0–5)	0.254
Face AIS—median (min–max)	0 (0–3)	0 (0–3)	0 (0–3)	0.67
Chest AIS—median (min–max)	3 (0–5)	3 (0–5)	4 (2–5)	0.647
Abdomen AIS—median (min–max)	2 (0–5)	2 (0–5)	1 (0–5)	0.273
Extremity—median (min–max)	2 (0–5)	2 (0–5)	2 (0–5)	0.422
External—median (min–max)	0 (0–2)	0 (0–2)	0 (0–1)	0.892
Pre-ICU days since trauma—median (min–max)	1 (0–14)	1 (0–5)	1 (0–14)	0.556
SAPS-II—mean (sd)	43.59 (13.16)	41.46 (14.03)	45.57 (12.21)	0.258
Died in ICU—n (%)	16 (29.63)	7 (26.92)	9 (32.14)	0.903
ICU length of stay—mean (sd)	26.98 (23.23)	14.81 (9.10)	38.28 (26.6)	<0.001
Mechanical ventilation—n (%)	52 (96.3)	25 (96.15)	27 (96.43)	1
Mechanical ventilation—mean (sd)	16.81 (16.19)	8.46 (7.67)	24.57 (18.18)	<0.001
CRRT—n (%)	11 (20.37)	3 (11.54)	8 (28.5)	0.224
Vasopressors—n (%)	40 (74.07)	17 (65.38)	23 (82.14)	0.274
Vasopressors—mean (sd)	4.09 (6.67)	2.92 (5.04)	5.18 (7.83)	0.211
Transfusion—n (%)	45 (83.33)	20 (76.92)	25 (89.28)	0.394
ECMO—n (%)	2 (3.70)	1 (3.85)	1 (3.57)	1
Tracheostomy—n (%)	25 (46.3)	9 (34.61)	16 (57.14)	0.166
Chest drain—n (%)	30 (55.55)	12 (46.15)	18 (64.28)	0.286
Surgery—n (%)	36 (66.67)	18 (69.23)	18 (64.28)	0.923
Chest—n (%)	2 (3.7)	1 (3.85)	1 (3.57)	1
Abdomen—n (%)	9 (16.67)	6 (23.08)	3 (10.71)	0.441
Neurosurgery—n (%)	7 (12.96)	3 (11.54)	4 (12.28)	1
Head and neck—n (%)	7 (12.96)	4 (15.38)	3 (10.71)	1
Spine—n (%)	3 (5.55)	0 (0)	3 (10.71)	0.228
Arms—n (%)	22 (40.74)	11 (42.31)	11 (39.28)	1
Plastic Surgery—n (%)	1 (1.85)	1 (3.85)	1 (3.57)	1

**Table 2:** Risk factors associated with the development of infection in patients colonized by CR-GNB.

	Overall Colonized (n = 28)		p-Value
	Not Infected n = 11 (39.3%)	Infected n = 17 (60.7%)	
age—mean (sd)	49.73 (25.45)	53.06 (16.85)	0.707
Charlson comorbidity index—median (min–max)	0 (0–4)	1 (0–3)	0.012
ISS—mean (sd)	28.72 (7.27)	32.23 (10.56)	0.308
Head and neck—median (min–max)	2 (0–4)	3 (0–5)	0.476
Face—median (min–max)	0 (0–2)	0 (0–3)	0.130
Chest—median (min–max)	3 (2–5)	4 (3–5)	0.997
Abdomen—median (min–max)	2 (0–5)	0 (0–4)	0.787
Extremity—median (min–max)	3 (0–4)	0 (0–5)	0.381
External—median (min–max)	0 (0–0)	0 (0–1)	0.421
Pre-ICU days since trauma—median (min–max)	1 ( 0–14)	1 (0–4)	0.058
SAPS II—mean (sd)	44.36 (14.89)	46.35 (10.55)	0.705
ICU length of stay—mean (sd)	24.82 (16.77)	47 (28.51)	0.016
Mechanical ventilation—n (%)	10 (90.91)	17 (100)	0.823
Mechanical ventilation—mean (sd)	13.54 (15.84)	31.7 (16.22)	0.008
CRRT—n (%)	1 (9.09)	7 (41.18)	0.159
Vasopressors—n (%)	8 (72.73)	15 (88.23)	0.588
Vasopressors—mean (sd)	1.09 (1.14)	7.82 (9.15)	0.008
Transfusion—n (%)	10 (90.91)	15 (88.23)	1
ECMO—n (%)	0 (0)	1 (5.88)	1
Tracheostomy—n (%)	5 (45.45)	11 (67.7)	0.539
Chest drain—n (%)	6 (54.54)	12 (70.59)	0.644
Surgery—n (%)	8 (72.73)	10 (58.82)	0.729
Time to colonization—mean (sd) days	10.09 (7.37)	8.94 (7.63)	0.695
Antibiotic before colonization—n (%)	11 (100)	17 (100)	N/A
Antibiotic before colonization—mean (sd) days	8.36 (4.29)	9.53 (4.58)	0.502
Piperacillin/tazobactam—n (%)	5 (45.45)	9 (52.94)	1
Pip. tazo—mean piperacillin/tazobactam (sd) days	1.64 (2.01)	3.12 (3.55)	0.172
Carbapenems—n (%)	1 (9.09)	5 (29.41)	0.419
Carbapenems—mean (sd) days	0.09 (0.30)	1.23 (2.11)	0.042
Cephalosporines—n (%)	1 (9.09)	1 (5.88)	1
Cephalosporines—mean (sd) days	1.27 (4.22)	0.29 (1.21)	0.469
Fluoroquinolones—n (%)	0 (0)	1 (5.88)	1
Fluoroquinolones—mean (sd) days	0 (0)	0.59 (2.42)	0.332
Glicopeptides—n (%)	4 (36.36)	7 (41.18)	1
Glicopeptides—mean (sd) days	2 (3.19)	1.65 (2.39)	0.757

## Data Availability

All data were reported in the manuscript, and the dataset will be available by special request to the corresponding author.

## Supplementary Materials

The following supporting information can be downloaded at: https://www.mdpi.com/article/10.3390/pathogens11111295/s1. Table S1. Patients at risk of colonization, n° of events, Survival Probability and 95%CI and Standard Error.
