# Supplementary material for: Clinical Impact of Colonization with Carbapenem-Resistant Gram-Negative Bacteria in Critically Ill Patients Admitted for Severe Trauma
Source: Pathogens. 2022 Nov 4;11(11):1295. doi: 10.3390/pathogens11111295 (PMC9695038; doi:10.3390/pathogens11111295)
Supplement: Supplementary file 1 [file pathogens-11-01295-s001.zip › pathogens-1963034-supplementary.pdf]

pathogens

MDPI

SUPPLEMENTARY MATERIALS

Table S1. Patients at risk of colonization, n° of events, Survival Probability and 95%CI and Standard Error.

Days	Patients at risk of colonization	n° of events	Free-from-colonization Probability	95%CI	SE
1	21	0	1.0000	1 – 1	0
5	20	1	0.95	0.86 – 1	0.05
10	13	9	0.52	0.35 – 0.79	0.35
15	9	4	0.33	0.18 – 0.61	0.10
20	3	5	0.09	0.02 – 0.36	0.06
